# Autologous Stem Cell Transplantation in elderly Acute Myeloid Leukemia

**DOI:** 10.4084/MJHID.2013.018

**Published:** 2013-02-16

**Authors:** Gert J Ossenkoppele, Jeroen JWM Janssen, Peter C Huijgens

**Affiliations:** Department of Haematology, VU. University Medical Center, Amsterdam, the Netherlands

## Abstract

Treatment outcome in elderly Acute Myeloid Leukemia (AML) is still very disappointing. Although complete remission rate is around 50–60% the 2 years survival is only in the magnitude of 10–20%. This is mainly due to an overrepresentation of adverse prognostic factors present in elderly AML. As relapses emerge from residual disease present after chemotherapy, intensification of treatment could emerge as a rational strategy. Intensification of chemotherapy by increasing the dose of anthracyclines or addition of gemtuzumab ozogamycin (Mylotarg) to standard chemotherapy indeed has proved to be of advantage in elderly AML. In younger AML autologous peripheral blood stem cell transplantation (AuPBSCT) as post remission treatment in comparison to intensive consolidation chemotherapy has been investigated in a few randomized studies. AuPBSCT showed reduced relapse rates with low non-relapse mortality rates. In elderly AML intensification by AuPBSCT also have been performed although randomized studies are lacking. Nevertheless, in the previous years various reports have suggested the potential utility of AuHSCT in AML of the elderly with encouraging results, albeit mostly in highly selected patients. Acceptable toxicity and a relatively low rate of transplant-related mortality has been notified. However relapses occurred which, irrespective of age, still remains the major cause of treatment failure of AuHSCT in AML. In this review we summarize the experience of AuPBSCT in elderly AML.

## Introduction

Accepted strategies to prevent relapse after achieving CR in AML are restricted to further intensive chemotherapy, allogeneic and autologous stem cell transplantation.^1^ Autologous hematopoietic cell transplantation (AuHSCT) has improved survival in a number of hematologic diseases, including lymphoma’s and multiple myeloma.^2^,^3^ However, the role of AuHSCT, although intensively studied is still a matter of debate in Acute Myeloid Leukemia(AML). Establishing the optimum role of auto transplants in AML has been challenging due to the heterogeneity of the disease and lack of results of randomized studies. Nonetheless, several trials have addressed this important question in de novo AML. Most of the studies have been performed in younger AML age groups and these will briefly be reviewed. Subsequently we will focus on the place of autologous stem cell transplantation in the elderly AML patients.

## Autologous Bone Marrow Transplantation in the Younger AML Patient

Eight randomized clinical trials compared the effect of autologous bone marrow transplantation with either no further treatment or additional intensive chemotherapy. 4–11 Only one of these studies showed a significantly better disease free survival (DFS) for patients treated with an autologous bone marrow transplantation^5^, while in none an advantage in overall survival was shown. All of these trials analyzed the outcome of the assigned post remission therapy on an intention to treat (ITT) basis. Interpretation of the true value of auto BMT was however hampered by the fact that only a minority of the patients allocated to auto BMT actually received the transplant due to early relapse, patient refusal, protocol violation or insufficient bone marrow harvest. A meta-analysis, pooling the data of these eight studies, also failed to show an overall survival (OS)benefit (effect size:0.84;1.02; 95% CI:0.91–1.15) for the patients consolidated with auto BMT although there was a statistically significant but modest improvement of DFS (effect size: 0.84, 95% CI:0.73–0.98).^12^ The discrepancy between DFS and OS is explained by an increased mortality due to toxicity in the transplant group, while the chance of successful rescue after relapse was better for patients in the control arms of the studies.

No randomized clinical trials comparing autologous with allogeneic transplantation have been performed. Instead, “genetic randomization” studies, using a donor versus no-donor analysis mostly showed a survival benefit for the allogeneic transplanted patients depending on age, risk profile and conditioning regimen.^13^ AuHSCT however compares favorably against allogeneic bone marrow transplantation in many ways. It avoids the, sometimes severe, sequelae of graft versus host disease(GVHD) and is associated with fewer life-threatening infections which are the most common causes of morbidity and mortality in allogeneic transplantations. Compared with repeated cycles of consolidation chemotherapy, the advantage of AuHSCT is shortening of treatment duration and a reduction of cumulative toxicity and repetitive periods of neutropenia.

## Peripheral Blood Stem Cell Transplantion in the Younger AML Patient

Before the introduction of growth factors as stem cell mobilizing agents, peripheral blood stem cell transplants (AuPBSCT) were cumbersome. Multiple steady state apheresis were required to achieve an adequate stem cell dose.^14^ After it became clear that granulocyte-colony stimulating factor (G-CSF), next to shortening the neutropenic period after chemotherapy, potently expanded the circulating CD34^+^38^−^haematopoetic stem cell pool in the peripheral blood,^15^ G-CSF, mostly in combination with chemotherapy became a standard method for stem cell mobilization, nearly completely replacing autologous bone marrow transplantation. Recently, plerixafor, a CXCR4 antagonist disrupting the binding of stem cells from the bone marrow environment was introduced for poorly mobilizing lymphoma and multiple myeloma patients.^16^ For stem cell mobilization in AML patients, the agent is not (yet?) approved, because of fear for possible co-mobilization of leukemic progenitors that then potentially could contaminate the apheresis product.

## Randomized Clinical Trials Investigating AuPBSCT in Younger AML Patients

Three studies in later years have investigated the role of AuPBSCT in AML in first complete remission (CR1). A small study (34 patients) by Tsimberidou et al showed an advantage of AuPBSCT over high dose cytarabine as consolidation treatment in terms of OS at 3 years (58% vs 46%) and failure free survival at 3 years (42% vs 33%), however significance was not reached possibly due to the small size of the study.^17^

In a large German Intergroup trial the two different postremission strategies (autologous SCT and maintenance chemotherapy) did not result in different OS, RD or RFS in the group of patients younger than age 60 or in any of the prognostic subgroups.^18^ However, results were slightly different in the Dutch-Belgian Hemato-Oncology Cooperative Group (HOVON) and Swiss Group for Clinical Cancer Research Collaborative Group (SAKK) leukemia cooperative groups who assessed the clinical benefit of AuPBSCT after high-dose cytotoxic therapy in a large multicenter study in 517 patients with AML in CR1 younger than 61 years after intensive anthracycline and cytarabine chemotherapy.^19^ AuPBSCT was prospectively compared with intensive consolidation chemotherapy with etoposide and mitoxantrone. Of patients randomized, more than 90% received their assigned treatment. The AuPBSCT group showed a markedly reduced relapse rate (58% vs 70%, P = .02) and better relapse-free survival at 5 years (38% vs29%, P =.065) with nonrelapse mortality of 4% versus 1% in the chemotherapy arm. Overall survival was however similar (44% vs 41% at 5 years), because of more opportunities for salvage with second-line chemotherapy and stem cell transplantation in patients relapsing in the chemotherapy arm.

The German Study Alliance Leukaemia (SAL) recently proposed a new prognostic score (PRT: post remission treatment score) useful for selection between autologous or allogeneic transplantation. The score is based on: age, %CD34+ blasts at diagnosis, FLT3-ITD mutant to wild type ratio, cytogenetic risk and de novo or secondary AML and defines three prognostic subgroups. Especially in the intermediate group AuPBSCT seems to be the treatment of choice, which was confirmed in an independent validation cohort.^20^

Minimal residual disease measurement before transplantation could also be regarded as an important factor influencing the choice of post remission treatment. Unfortunately, in the autologous setting there is few data available. In an Italian study, MRD-negative patients had a good outcome regardless of the type of transplant they received. However, in the MRD-positive group, AuPBSCT did not improve prognosis and alloSCT represented the primary option.^21^

Overall treatment related mortality is indeed clearly reduced in the AuPBSCT era as compared to the bone marrow derived stem cell transplantations. For selected patients it can be regarded as an effective treatment reducing relapse and in comparison to repeated cycles of HiDAC it shortens the duration of treatment. The risk of relapse should be counterbalanced against the non-relapse mortality of allogeneic transplantation. 22

## Purging

Although contamination of the graft may be regarded as a reflection of persistent systemic disease, there is clear evidence that leukemic cells in the graft may contribute to relapse. Elimination of residual leukemic cells from the graft by purging techniques may therefore be a rational approach. Data concerning this issue must be derived from phase II studies, because no randomized phase III trials have been performed. In most studies, purging was performed by ex-vivo addition of 4-hydroxy-cyclofosfamide (4-HC; mafosfamide) to the graft.^23^ In a retrospective case control study by the ABMTR in 294 patients a decrease in the relapse rate from 42% to 34% in CR1 patients with 4-HC purged grafts was noticed, with an improvement in 3 year Leukemia Free Survival from 31 to 56%.^24^ Despite these promising results, 4-HC the data have never been regarded convincing enough for approval of the drug by FDA or EMA. One of the major drawbacks of the drug is a long delay in engraftment. Another purging approach is the use of monoclonal antibodies directed against CD14 and CD15, as well as the incubation with IL2. Both have also shown to be feasible.^25^,^26^ Nevertheless, no stem cell graft purging method is currently accepted as standard treatment.

## AML in Elderly

Treatment for AML results in a CR of 70–80% in patients below 60years and around 40% long term survival. Outcome in older patients is much less satisfactory with a 2 years survival of 10–20%. This is due to an overrepresentation of adverse prognostic factors and the inability to deliver intensive therapy in these older patients.. As relapses emerge from residual disease present after chemotherapy, intensification of treatment is a rational approach.^27^ Even in the elderly AML population, this proved feasible. Increasing the dose of daunorubicin to 90 instead of 45 mg/m^2^ was tolerated quite well and resulted in a better outcome.^28^

Also, the addition of gemtuzumab ozogamycin (GO, Mylotarg) to standard induction treatment showed to be advantageous in terms of survival in both the French ALFA group and the British MRC AML trial group.^29^,^30^ Due to the lower dosages of GO that were used, toxicity was less in comparison to the first clinical trials in which especially liver toxicity was prominent.

Much debate is ongoing around the question whether intensive treatment should be offered to the elderly patient with AML. Data from the Swedish registry showed that most patients up to 80 years actually tolerate intensive treatment, despite deteriorating organ functions, and that response rates are acceptable for those who receivedit.^31^ Patients not given intensive treatment, mostly received supportive palliative care only, or oral hydroxyurea or thioguanin, whereas subcutaneous low dose cytarabine(20 mg/m^2^ twice daily) was rarely given. It was shown that in every age cohort of patients the early death rate within 8 weeks from diagnosis was lower with intensive treatment than with palliation. This was not solely due to selection of better patients to intensive treatment. Even patients with high risk genetics seemed to benefit from intensive treatment, although survival remained limited. Certainly, there is room for improvement to increase response rates and reduce toxicity, but even more to prolong remission duration and thereby provide a possibility for a better life expectancy with quality. Age on its own should not be regarded as reason to withhold intensive treatment.

If intensive post remission intensification therapy is of benefit for the elderly patient with AML is however unsolved. Various attempts for intensification by increasing doses and/or cycles of chemotherapy after CR has been reached have failed. Recently, Burnett et al showed that 1 versus 2 courses post remission were equally effective. Whether stem cell transplantation, be it allogeneic or autologous, can prevent relapse and improve survival of the elderly patient with AML is currently unclear.^32^

## Allogeneic Transplantation in the Elderly

Myeloablative conditioning regimens as consolidation treatment are not feasible for the vast majority of elderly patient with AML due to the associated toxicities. Fortunately, the development of reduced intensity (RIC) conditioning transplant regimens has paved the way for allotransplants in the elderly age cohort. Numerous regimens have been described varying from truly non-myeloablative (single dose TBI) to those that cause profound cytopenia (Fludarabine/Busulphan) requiring blood and platelet support. Various, relatively small and uncontrolled, studies showed that this form of transplantation is feasible in older AML patients with encouraging outcomes. Results may however be flattered due to selection of patients*33–36* As an example*,* Estey et all calculated that from a cohort of 259 elderly patients, only 5% actually received the allogeneic transplant.^37^ An ongoing ELN study in Europe is currently randomizing patients above 60 years with AML in CR1 to either receive a RIC transplant or standard treatment. Results of this important study are awaited eagerly.

## Autologous Transplantation in the Elderly

Prospective randomized studies in older AML patients comparing high dose cytarabine based intensive consolidation with autologous stem cell transplantation or allogeneic stem cell transplantation (allo-SCT) are lacking. This is mainly due to the difficult accrual of elderly patients in either HiDAC consolidation or stem cell transplantation trials. Nevertheless, in the previous years various reports have suggested the potential utility of AuHSCT in AML of the elderly with encouraging results, albeit mostly in highly selected patients. In particular, the use of peripheral blood stem cells (PBSC) as stem cell source has proven to be feasible. Acceptable toxicity and a relatively low rate of transplant-related mortality was seen without affecting the rate of relapse which, irrespective of age, still remains the major cause of treatment failure of AuHSCT in AML.

Several groups have reported results of AuHSCT for elderly AML patients over the age of 60(a selection of studies is presented in Table 1). Cahn et al compared 111 AML patients in CR1 over the age of 50 years treated with AuHSCT with a cohort of 786 patients below the age of 50. Stemcells were bone marrow derived and the various conditioning regimens were either chemotherapy or radiotherapy based. LFS at 4 years for patients aged over 50 years was 34% ±5%, compared with 43%± 2% for patients less than50 years of age, with an overall survival probability of35% ± 6% and 48% ± 2%, respectively. Despite the significant difference in LFS and OS between the groups, no difference was observed in terms of relapse (52% ±7%versus 50% ± 2%, respectively).^39^

Gorin et al reported the outcome of 193 elderly patients transplanted between 1984–1998.^40^ The stemcell source was peripheral blood in 128, bone marrow in 51 and a combination in 14 patients. 147 patients were transplanted in CR1by the use of various conditioning regimens (34 patients received TBI). Treatment related mortality (TRM)was 15%±4%, leukemia free survival (LFS) was 36%±5% and overall survival(OS) 47%±5%at 3 years. OS was significantly improved in the years after 1995. Remarkably, the patients who received bone marrow derived stem cells had a lower rate of relapse than those who received peripheral blood stem cell grafts: 44% ±11% versus 63% ±6% respectively.

In another elderly AML study of the CETLAM group, from 258 registered patients, 135 were enrolled for intensive treatment, of which ultimately only 16(27%) received an AuPBSCT (see table 1).^41^ Lashkari et al treated 27 patients aged over 60 years with AML in CR1 with PB stem cells after a conditioning regimen of Cy/TBI. Actuarial LFS and OS at 3 years was 25%±9% and 28%±9%, respectively.^42^

Again demonstrating the level of selection of elderly patients who received autoSCT, the EORTC-GIMEMA experience in elderly AML was reported by Thomas et al.^43^ Only 61 patients of an initial cohort of 787 randomized patients were scheduled for transplantation from which in the end only 35 actually received an AuPBSCT. The reasons for not being transplanted were inadequate harvest (21 patients), early relapse(3) and refusal(2). However, the survival outcome in patients transplanted as compared to those scheduled for but in fact not transplanted seemed to be identical.

Ferrara et al. treated 40 patients (median age, 67 years) with AuPBSCT and noted that the median LFS and overall survival from diagnosis were 13 and 22 months, respectively.^44^ They used an alternative conditioning regimen consisting of 2 days continuous infusion of idarubicin at 20 mg/m^2^/day, followed by 3 days oral or intravenous busulphan (4 mg/kg/day). No treatment related mortality was noted. The results in the intermediate risk categories were much better as compared to the adverse cytogenetic risk categories.

Lemoli et al used a dose adjustment for a busulphan/melphalan regimen for patients with AML in CR1 above 60 years. DFS for patients above 55 years was 50% and OS 50 % with a median follow up of 31 months.^45^ This compares favorably with what is usually observed with chemotherapy alone. Again, one has to consider that the transplanted elderly AML patient is highly selected, introducing significant bias.

Finally a retrospective EBMT study compared the outcome of 361 allo-RIC transplants with 1369 AuPBSCT in patients with AML over 50 years.^46^ LFS(RR1.22, p=0.02 and OS (RR1.32, p=0.005) were superior in the RIC patients. However inCR1 no survival benefit was present due to the fact that the lower relapse rate was counterbalanced by a significantly increased non relapse mortality. The outcome data of the 1152 patients transplanted in CR1 are given in table 1.

## Mobilization in the Elderly

The influence of age on mobilization of peripheral blood stem cells in AML was investigated by Ferrara et al who performed a retrospective analysis and compared patients below and above 60 years of age.^38^ The median number of collected CD34+ cells was comparable between the two groups. A successful mobilization rate (CD34+ cells of >2×10^6^/kg) of 80% vs 87% could be shown. Also, the median numbers of CD34+ cells harvested and the number of days of apheresis necessary to collect enough stem cells was independent of age. The authors concluded that age has no impact on mobilization and collection of PBSCs in AML.

## Conclusion

No randomized clinical studies investigating the role of AuPBSCT in elderly AML have been performed. The published data in AML CR1 represent highly selected patients that certainly are not representative for all AML patients above the age of 60. The published studies show that in selected patients AuPBSCT is feasible with relative acceptable toxicity and results in encouraging outcomes for those that actually received the transplant. Whether this is really of advantage for the total group of patients that can be offered intensive post remission treatment has to be solved in randomized trials. We however doubt whether any trial group is willing to set out such a study. Most AML trial groups are probably more interested in the development of new targeted drugs in combination with standard chemotherapy and immune modulation(allo-RIC, NK cell therapy, dendritic cell vaccination etc.) after achieving CR.

## Figures and Tables

**Table 1:** Selected non randomized studies on autologous stem cell transplantation in elderly AML

	No of Pts	Median Age (limits)	Stem cell source	Conditioning regimen	TRM	Outcome DFS/LFS	Outcome OS
**Cahn199539**	111	53 (50–63)	BM	Various	28%±5%	34%±5% at 4 yrs	35%±6% at 4 yrs
**Gorin 200040**	193	63 (60–75)	PB: 128 BM: 51 PB+BM:14	Various TBI: 34Pts	15%±4%	36%±5% at 3 yrs	47%±5% at 3 yrs
**Oriol 200441**	16*	64 (61–70	PB	Cy/TBI Bu/Cy	3/16	39% at 2 yrs	NA
**Lashkari 200642**	27	65 (60–71)	PB	Cy/TBI	1/27	25%±9% at 3 yrs	28%±9% at 3 yrs
**Thomas 200743**	35**	64 (60–69)	PB	BVAC	NA	28% at 3 yrs	39% at 3 yrs
**Ferrara 200944**	40	67 (61–77)	PB	Ida c.i./Bu	0%	Median: 13 months ± 40 % at 3 yrs***	Median :22 Months ± 40% at 3 yrs***
**Herr 200746**	1152****	57***** (50–78)	PB	Variuos	NRM at 2 yrs: 8%±1%	42%±2% at 2 yrs	52%±2% at 2 yrs

* from an initial cohort of 258 patients from which 135 received intensive induction.

** 59 patients in CR1 with PS 0–1 out of a initial randomized cohort of 787patients were scheduled for AuPBSCT of which in only 35 a AuPBSCT was performed.

*** estimated from the graphs in the manuscript

**** Patients in CR1

***** CR1, CR2 and advanced patients

Abbreviations: DFS: disease free survival, LFS: leukemia free survival, OS: overall survival, BM: bone marrow, c.i.: continuous infusion, PB: peripheral blood, NRM: non relapse related mortality, Cy: Cyclofosfamide, Bu: busulphan, TBI: total body irradiation, BVAC: carmustine, amsacrine, etoposide, cytarabine.
